# The i-Laparotrainer

**DOI:** 10.1308/rcsann.2014.96.1.76a

**Published:** 2014-01

**Authors:** T Antonios, H Abboudi, K Amin

**Affiliations:** Brighton and Sussex University Hospitals NHS Trust,UK

Laparoscopic training is fundamental to general surgery. In order to aid acquisition of basic laparoscopic skills, we suggest using the iPad® (Apple, Cupertino, CA, US). iPad® ownership among doctors is increasing, with a survey from 2012 showing 31% own or use an iPad® at work.^1^

**Figure 1:**
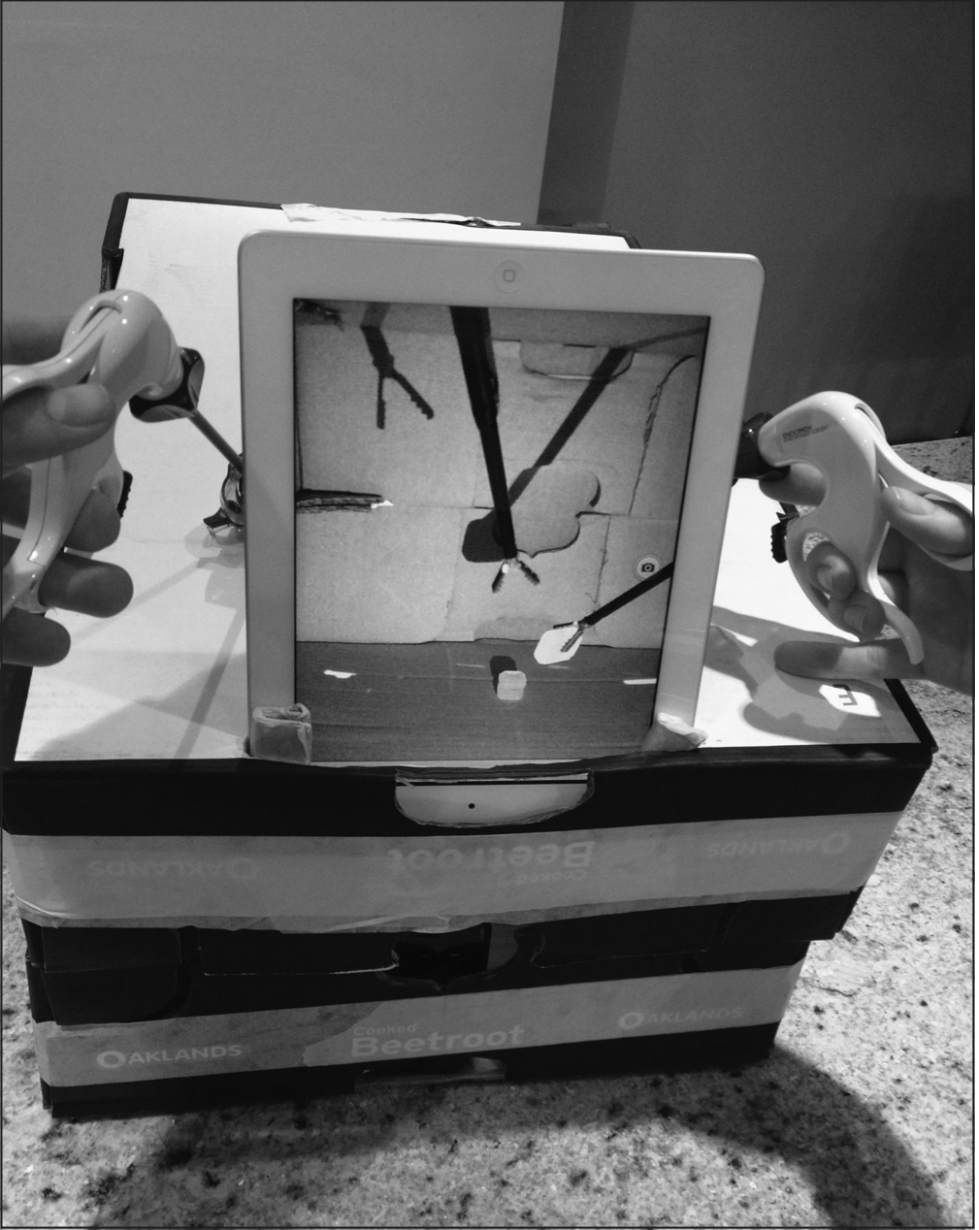
Using the iPad© as a basic laparoscopic trainer

The iPad® is incorporated into a box with the backlight illumination function turned on and the high resolution camera reversed so that the viewing portal is facing the trainee (Fig 1). This simple method can enable medical students and junior doctors to acquire the basic skills prior to embarking on expensive courses by using a resource that many people already own.
